# Effect of heating and glycation on the allergenicity of Ara h 2/6

**DOI:** 10.1186/2045-7022-1-S1-O13

**Published:** 2011-08-12

**Authors:** Fany Blanc, Yvonne Vissers, Per Stahl Skov, Phil Johnson, Clare Mills, Harry Wichers, Karine Adel-Patient

**Affiliations:** 1 INRA, Unite d'Immuno-Allergie Alimentaire, Jouy-en-Josas, France; 2 Wageningen University, Cell Biology and Immunology, Wageningen, Netherlands; 3 RefLab ApS, Copenhagen, Denmark; 4 Institute of Food Research, Norwich, UK

## Aim

To study the effect of heating and glycation on the IgE-binding properties and biological activity of 2S albumins (Ara h 2/6) from peanut.

## Methods

Native Ara h 2/6 was purified from raw peanuts and heated in solution (15 min, 110°C) in either the presence or absence of glucose, or purified from roasted peanut. Using PBMC and sera from peanut allergic patients the cellular proliferative potency, IgE reactivity (reverse EAST inhibition) and functionality (basophils activation) of allergens were assessed.

## Results

Heating Ara h 2/6 at 110°C resulted in extensive denaturation whilst Ara h 2/6 extracted from roasted peanut retained its native conformation. Allergen stimulation of PBMC from peanut allergic patients induced proliferation of mainly CD4+ T-cells and induction of Th2 cytokine secretion which was unaffected by thermal processing. IgE reactivity and functionality of Ara h 2/6 was decreased by heating. Whilst heating-glycation further reduced the IgE binding capacity of the proteins, it moderated their loss of histamine releasing capacity. Ara h 2/6 purified from roasted peanut demonstrated the same IgE reactivity as unheated, native Ara h 2/6.

## Conclusion

Although no effect of processing on T-cell reactivity was observed, heat induced denaturation and reduced the IgE reactivity and functionality of Ara h 2/6; Ara h 2 and 6 purified from roasted peanut retained the structure and IgE reactivity of the native protein. This study further demonstrates the effect of thermal treatment on the allergenicity of peanut allergens.

